# Exploratory observational study of the association between bacterial L-form detection and intrauterine infection in pregnant women with placenta previa and bleeding

**DOI:** 10.3389/fmed.2026.1726426

**Published:** 2026-05-01

**Authors:** Sue Jiang, Xiaoqiong Li, Yunbo Wei, Xiaoxiao Zheng

**Affiliations:** 1 Pregnancy Health Care Department, Huai'an Maternal and Child Health Care Hospital Affiliated to Yangzhou University, Huai'an, China; 2 Department of Obstetrics, Huai'an Maternal and Child Health Care Hospital Affiliated to Yangzhou University, Huai'an, China; 3 Emergency Medicine Department, Huai'an Maternal and Child Health Care Hospital Affiliated to Yangzhou University, Huai'an, China

**Keywords:** bacterial L-form, chorioamnionitis, intrauterine infection, placenta previa, pregnancy outcomes, vaginal bleeding

## Abstract

**Objective:**

This study aimed to conduct an exploratory observational analysis to assess the association between bacterial L-form detection and intrauterine infection in pregnant women with placenta previa and bleeding, and to describe its potential relationship with maternal and fetal outcomes.

**Methods:**

A total of 120 patients with placenta previa and bleeding were enrolled and divided into an L-form positive group (*n* = 80) and an L-form negative group (*n* = 40). The L-form positive group was further subdivided into patients with positive detection at admission (*n* = 40) and before surgery (*n* = 40). Maternal peripheral blood, vaginal secretions, placental tissue, umbilical cord tissue, neonatal umbilical cord blood, and neonatal peripheral blood were collected for bacterial L-form culture using hypertonic osmoprotective media. Contamination control procedures, parallel negative controls, duplicate inoculation, and predefined quality control procedures were applied throughout culture and identification. Clinical data and placental pathology were analyzed, and unadjusted odds ratios with 95% confidence intervals were calculated as descriptive measures of association.

**Results:**

The L-form positive group had higher rates of multiple miscarriages (unadjusted OR = 2.67, 95% CI: 1.10–6.47), uterine cavity procedures (unadjusted OR=2.39, 95% CI: 1.10–5.18), and previous cesarean sections (unadjusted OR = 2.68, 95% CI: 1.07–6.73) than the L-form negative group. This group also showed lower gestational age and birth weight, together with higher rates of neonatal pneumonia (32.5% vs. 7.5%, unadjusted OR = 5.93, 95% CI: 1.67–21.04), chorioamnionitis (35.0% vs. 10.0%, unadjusted OR = 4.85, 95% CI: 1.57–14.95), and neonatal antibiotic use (40.0% vs. 12.5%, unadjusted OR = 4.67, 95% CI: 1.67–13.04). Exploratory susceptibility testing suggested low sensitivity to cell wall-active antibiotics and relatively higher sensitivity to polymyxin, rifampin, protein synthesis inhibitors, and cephalosporins. L-form detection in umbilical cord blood and neonatal peripheral blood was more frequent in the L-form positive group, and bacterial L-form detection showed descriptive correlations with lower gestational age, lower birth weight, pneumonia, neonatal mortality, and more severe chorioamnionitis.

**Conclusion:**

In this exploratory observational study, bacterial L-form detection was associated with markers of intrauterine infection in pregnant women with placenta previa and bleeding. These findings should be interpreted cautiously because the analyses were unadjusted and the microbiological workflow remains exploratory.

## Introduction

Placenta previa is one of the most challenging and serious complications of pregnancy. Its core characteristic is abnormal attachment of the placenta to the lower segment of the uterus, partially or completely covering the internal os of the cervix, with a position below the presenting part of the fetus. This unique anatomical change can lead to unexplained, painless recurrent vaginal bleeding from late pregnancy to postpartum, posing a continuous threat to the health and safety of both mother and baby (1–3). Due to repeated blood loss leading to impaired immune function, pregnant women with placenta previa and bleeding are at high risk for intrauterine infection. Intrauterine infection further triggers a series of adverse pregnancy outcomes, including fetal growth restriction, preterm birth, premature rupture of membranes, and neonatal infection, forming a vicious cycle of “bleeding–impaired immunity – infection–adverse outcomes” (4, 5). In recent years, with the accumulation of high-risk factors such as a history of cesarean section, uterine cavity procedures, advanced maternal age, multiple pregnancies, smoking, and assisted reproductive technology (ART), the incidence of placenta previa has shown a steady upward trend, and the clinical management challenges associated with it have continued to increase (3, 6).

Clinical studies have documented the association between placenta previa and intrauterine infection: Park et al. (7) found that 19.0% of cases with placenta previa and intact membranes had placental inflammation, and 16.7% had intra-amniotic inflammation, clearly identifying placenta previa as a major risk factor for neonatal infection. Another study noted that among pregnant women with placenta previa who experienced spontaneous vaginal bleeding, 17.9% had subclinical amniotic cavity infection and/or inflammation, suggesting that vaginal bleeding may be an important indicator of underlying infection, and such occult inflammation is a high-risk factor for preterm birth (8). These findings challenge the previous understanding of placenta previa bleeding as solely due to mechanical injury, highlighting the critical role of infection in disease progression. Bacteria are the primary pathogens responsible for intrauterine infections, and their special variant form—bacterial L-forms—has increasingly drawn attention for their potential pathogenicity (9). Bacterial L-forms are variants formed when bacterial cell walls are lost or defective due to physical, chemical, or biological factors. Their core hazard lies in their ability to evade antibiotic attacks and immune clearance by the host, leading to persistent or recurrent infections. Additionally, they are difficult to detect via conventional bacterial culture, making them a “blind spot” in clinical diagnosis (10, 11). The role of bacterial L-forms in chronic infections has been documented (10), but research on their association with intrauterine infections in pregnant women with placenta previa and bleeding remains limited, leaving unresolved the clinical paradox of “negative conventional infection markers yet severe neonatal infections.”

Based on the above background, this study focuses on the interaction between the latent pathogenicity of bacterial L-forms and the susceptibility to infection in patients with placenta previa and bleeding. This study aimed to conduct an exploratory observational analysis to assess the association between bacterial L-form detection and intrauterine infection in pregnant women with placenta previa and bleeding, to describe the distribution patterns and microbiological characteristics of bacterial L-forms, and to explore their potential relationship with maternal and neonatal outcomes.

## Materials and methods

### Study population

This study was approved by the Ethics Committee of Huai'an Maternal and Child Health Care Hospital Affiliated to Yangzhou University (Approval No. HAFY-2021-KY-042). Informed consent was waived due to the retrospective nature of this study, and all methods were performed in accordance with relevant guidelines and regulations. A total of 120 patients hospitalized at the hospital from July 2021 to September 2023 who were diagnosed with placenta previa accompanied by bleeding were selected as the study population. Inclusion criteria: ① Confirmed by ultrasound as placenta previa (the placenta is implanted in the lower segment of the uterus or covers the internal os of the cervix, located anterior to the presenting part of the fetus); ② Patients experienced definite vaginal bleeding during pregnancy, and other causes of bleeding, such as trauma or cervical lesions, were excluded; ③ Single pregnancy; ④ Complete clinical records and follow-up data available. Exclusion criteria: ① Patients with severe liver or kidney dysfunction, malignant tumors, or other underlying diseases; ② Patients with uncontrolled severe complications of pregnancy (such as severe preeclampsia or diabetic ketoacidosis during pregnancy); ③ Recent (within 1 month) use of immunosuppressive agents; ④ Fetal severe malformations (e.g., anencephaly, severe cardiac malformations) or chromosomal abnormalities (confirmed by amniocentesis or non-invasive DNA testing); ⑤ Incomplete clinical data or withdrawal from the study.

### Grouping method

Grouping was based on the timing of bacterial L-form detection. At admission, peripheral blood and secretions from the upper third of the vagina were collected for bacterial L-form culture. Patients with positive culture results were assigned to the admission-positive L-form subgroup (*n* = 40). Patients with negative results entered an observation period during which maternal symptoms and infection markers were closely monitored. 24 to 48 h before surgical termination of pregnancy, the same samples were collected again for culture. Patients with positive results at that time were assigned to the preoperative-positive L-form subgroup (*n* = 40), whereas those with persistently negative results constituted the L-form negative group (*n* = 40). For the primary analysis, the two L-form positive subgroups were combined as the L-form positive group (*n* = 80). Patients in the L-form positive group received antibiotic treatment according to the exploratory susceptibility results and clinical judgement, whereas the L-form negative group received routine broad-spectrum prophylactic treatment according to institutional practice.

### Sample collection

Sample collection upon admission and prior to surgery: Collect peripheral blood from the pregnant woman (approximately 1 ml from the elbow vein) using an EDTA anticoagulant tube. Gently invert and mix 5–8 times to avoid hemolysis. Collect secretions from the upper third of the vagina using a sterile cotton swab, rotating and wiping the lateral wall of the upper vagina 3–5 times to ensure the swab fully contacts the secretions. Prioritize collecting secretions near the cervical os that are accompanied by endometrial blood. Place the swab into a sterile saline tube and seal for storage.

Sample collection during cesarean section: Collect placental tissue (avoid calcified and necrotic areas, approximately 1.5 cm × 1.5 cm × 1 cm in size); Collect umbilical cord tissue by cutting approximately 1 cm from the fetal end, removing the umbilical arteries and veins, then chopping the remaining tissue into pieces of 0.5 mm^3^−2 mm^3^ and placing them in a sterile container; Collect umbilical cord blood from live-born newborns. Immediately after cord clamping, draw approximately 1 ml from the umbilical vein using an EDTA anticoagulant tube. If the newborn is transferred to another department for treatment, collect approximately 1 ml of peripheral blood from the newborn within 24 h of transfer using an EDTA anticoagulant tube. All sample collection procedures were performed under strict aseptic conditions with sterile technique. The sampling site was disinfected prior to sampling, and contamination was avoided during the process. For cord blood and neonatal peripheral blood collection, the skin was disinfected with 70% isopropyl alcohol followed by 2% chlorhexidine gluconate, and sterile needles and collection tubes were used. After sample collection, the sample information (including patient ID, sampling time, sample type, etc.) was immediately labeled, and the sample was sent to the laboratory for processing within 2 h. Additionally, the placenta of all enrolled participants was sent to the pathology department for examination to determine whether chorioamnionitis was present.

### Bacterial L-form culture and identification

Bacterial L-form culture was performed using a hypertonic osmoprotective medium, following established protocols for cell wall-deficient bacteria (12, 13). The enrichment medium contained beef extract, peptone, sodium chloride, glucose, horse serum, and agar, with osmotic pressure adjusted by sucrose and magnesium sulfate. Before use, each batch of medium underwent sterility assessment and growth-promotion testing. Staphylococcus aureus ATCC 25923 and Escherichia coli ATCC 25922 were used as parallel quality-control strains after osmotic adaptation to verify that the medium supported the expected atypical growth patterns, while uninoculated medium and sterile saline processed through the same workflow served as negative controls.

Culture procedure: Sample collection, inoculation, incubation, and reading were performed by trained technicians using a standardized workflow in a biosafety cabinet. The quality of each culture bottle was checked before inoculation, sample identifiers were verified, and bottle stoppers were disinfected with iodophor and 75% ethanol. Each clinical specimen was inoculated in duplicate, and a matched negative-control tube was opened and processed with every batch. Samples were incubated at 35 °C for 72 h and then subcultured onto hypertonic solid medium. Plates were observed independently by two microbiology technicians at 24 h intervals for up to 7 days. When colony morphology was equivocal, repeat subculture was performed from the original enrichment broth. A culture result was considered reportable only when duplicate inoculations showed concordant findings and the corresponding negative control remained sterile.

Result interpretation: Positive growth (+) was defined as turbidity in the upper layer of the culture bottle, filamentous suspended particles, wall-adhering growth, or characteristic “fried-egg” or granular colonies on hypertonic solid medium confirmed on repeat observation. Negative growth (–) required the absence of these findings in duplicate cultures together with sterile negative controls. To improve reliability, all positive cultures were re-read by a senior microbiologist, and discordant results were resolved by repeat inoculation from stored specimens when residual material was available.

### Antibiotic susceptibility testing

Exploratory antibiotic susceptibility testing was performed on L-form positive bacterial samples using the disk diffusion method, following the operational standards recommended by the clinical microbiology laboratory. Because validated breakpoints for L-form bacteria are lacking, these results were used only as exploratory microbiological descriptors and not as definitive guidance for efficacy. Each run included parallel quality-control strains (S. aureus ATCC 25923 and E. coli ATCC 25922), duplicate plates, and a medium-only negative control to monitor contamination and batch consistency.

### Placental pathology examination

All placental tissue was sent to the pathology department, fixed in 10% neutral formaldehyde for 24–48 h, routinely dehydrated, paraffin-embedded, sectioned into 4 μm thick slices, and stained with hematoxylin and eosin (HE). Two senior pathologists (with over 10 years of diagnostic experience) reviewed the slides using a double-blind method to determine the presence of chorioamnionitis. The diagnosis was based on the presence of polymorphonuclear leukocyte infiltration in the chorioamniotic membranes. Inflammatory cell infiltration was classified into mild (limited to the superficial layer of the chorion, grade 1) and moderate to severe (extending into the deep layers of the chorion or amnion, accompanied by villous edema, necrosis, etc., grades 2–3) according to established histopathological criteria. If the two pathologists' diagnoses differed, a consensus was reached through joint review and discussion.

### Clinical data collection

Record detailed clinical data for all mothers and infants included in the study, including maternal age, height, weight, parity, number of prenatal visits, comorbidities (gestational hypertension, diabetes, and anemia), and risk factors (history of multiple miscarriages, history of uterine procedures, and history of cesarean section). For offspring, record gestational age at birth, birth weight, and clinical outcomes. Neonatal pneumonia was diagnosed based on the presence of respiratory symptoms (tachypnea, grunting, nasal flaring, and chest retractions), abnormal chest radiograph findings (infiltrates, consolidation, or air bronchograms), and/or positive blood culture or elevated inflammatory markers (C-reactive protein >10 mg/L or procalcitonin >0.5 ng/mL within 72 h of birth). Neonatal mortality was defined as death within the first 28 days of life.

### Statistical analysis

Data were analyzed using GraphPad Prism 9.50 software (GraphPad Software Inc., San Diego, CA, USA) and SPSS 21.0 statistical software (SPSS, Inc., Chicago, IL, USA). Sample size estimation was not performed because this was an exploratory retrospective study. Continuous data were tested for normality using the Shapiro–Wilk test. Data conforming to normal distribution are presented as mean ± standard deviation and compared using the independent-samples *t* test; non-normally distributed data are presented as median (interquartile range) and compared using the Mann–Whitney U test. Categorical variables are expressed as counts and percentages and compared using the chi-square test or Fisher's exact test, as appropriate. Unadjusted odds ratios with 95% confidence intervals were calculated for major binary outcomes as descriptive association measures. Spearman's correlation analysis was used to describe associations between bacterial L-form detection and intrauterine infection-related indicators. All tests were two-sided, and *P* < 0.05 was considered statistically significant.

## Results

### Baseline characteristics of study subjects

A total of 120 patients with placenta previa and bleeding were included in this study. They were divided into an L-form positive group (bacterial L-form positive, *n* = 80) and a L-form negative group (bacterial L-form negative, *n* = 40) based on the results of bacterial L-form detection. The L-form positive group included Trial Group 1 (*n* = 40) with positive detection at admission and Trial Group 2 (*n* = 40) with positive detection before surgery. There were no statistically significant differences between the two groups in terms of age, height, weight, parity, number of prenatal visits, and comorbidities (gestational hypertension, diabetes, and anemia) at baseline (*P* > 0.05) (Table 1), indicating comparability in these variables. However, analysis of high-risk factors revealed significant baseline differences: in the L-form positive group, 32 cases (40.00%) had a history of multiple miscarriages, 45 cases (56.25%) had a history of uterine cavity procedures, and 29 cases (36.25%) had a history of cesarean section; while the L-form negative group had 8 cases (20.0%), 14 cases (35.00%), and 7 cases (17.5%), respectively. The unadjusted proportions of multiple miscarriages (OR = 2.67, 95% CI: 1.10–6.47, *P* = 0.029), history of uterine cavity procedures (OR = 2.39, 95% CI: 1.10–5.18, *P* = 0.028), and history of cesarean sections (OR = 2.68, 95% CI: 1.07–6.73, *P* = 0.035) were significantly higher in the L-form positive group than in the L-form negative group (Table 1). These baseline differences represent important potential confounders that limit interpretation of subsequent outcome comparisons.

**Table 1:** Baseline characteristics of study subjects.

Variable	L-form positive group (*n* = 80)	L-form negative group (*n* = 40)	Unadjusted OR (95% CI)	t/Z/χ^2^	*P*
Age (years)	28.89 ± 3.97	28.25 ± 4.30	–	0.807	0.421
Height (cm)	160.10 ± 4.69	159.70 ± 4.87	–	0.409	0.684
Weight (kg)	62.64 ± 7.35	62.88 ± 7.82	–	0.163	0.871
Number of births (times)	1 (1, 2)	1 (1, 2)	–	−0.469	0.635
Number of prenatal checkups	7.23 ± 2.19	7.18 ± 2.31	–	0.116	0.908
Comorbidities (*n*, %)					
Pregnancy-induced hypertension	8 (10.00%)	5 (12.50%)	0.78 (0.24–2.53)	0.173	0.678
Diabetes	5 (6.25%)	2 (5.00%)	1.27 (0.24–6.77)	0.076	0.783
Anemia	21 (26.25%)	12 (30.00%)	0.83 (0.36–1.92)	0.188	0.665
High-risk factors (*n*, %)					
History of multiple miscarriages	32 (40.00%)	8 (20.00%)	2.67 (1.10–6.47)	4.800	0.029
History of intrauterine procedures	45 (56.25%)	14 (35.00%)	2.39 (1.10–5.18)	4.818	0.028
History of cesarean section	29 (36.25%)	7 (17.50%)	2.68 (1.07–6.73)	4.464	0.035

Count data are expressed as counts and percentages; continuous data following normal distribution are expressed as mean ± standard deviation; non-normally distributed data are expressed as median (interquartile range). Odds ratios are unadjusted and should not be interpreted as independent associations. *P* < 0.05 indicates statistical significance.

### Comparison of offspring outcomes

A comparative analysis of key outcome measures for the two groups of offspring was conducted. The results showed that the average gestational age of the offspring in the L-form positive group was 32.00 (31.00, 33.00) weeks, significantly lower than the 35.00 (33.00, 36.00) weeks in the L-form negative group. The average birth weight of the L-form positive group offspring was (2,300 ± 156.90) g, significantly lower than the L-form negative group's (2,535 ± 171.40) g, with all differences being statistically significant (all *P* < 0.001). In terms of adverse outcomes, the pneumonia infection rate in the L-form positive group was 32.5% (26/80), higher than the 7.5% (3/40) in the L-form negative group (unadjusted OR = 5.93, 95% CI: 1.67–21.04, *P* = 0.003). Additionally, the neonatal mortality rate in the L-form positive group was 10.00% (8/80), higher than the 2.5% (1/40) in the L-form negative group (unadjusted OR = 4.33, 95% CI: 0.52–36.00), but the difference was not statistically significant (*P* = 0.141), likely due to insufficient statistical power for this rare outcome (Table 2). It is important to note that these unadjusted comparisons do not account for the significant baseline differences in uterine trauma history between groups, which are themselves known risk factors for adverse pregnancy outcomes.

**Table 2:** Comparison of offspring outcomes.

Offspring outcome measures	L-form positive group (*n* = 80)	L-form negative group (*n* = 40)	Unadjusted OR (95% CI)	t/Z/χ^2^	*P*
Average gestational age (weeks)	32.00 (31.00, 33.00)	35.00 (33.00, 36.00)	–	−5.165	<0.001
Average weight (g)	2,300 ± 156.90	2,535 ± 171.40	–	7.509	<0.001
Pneumonia infection rate (*n*, %)	26 (32.50%)	3 (7.50%)	5.93 (1.67–21.04)	9.094	0.003
Neonatal mortality rate (*n*, %)	8 (10.00%)	1 (2.5%)	4.33 (0.52–36.00)	2.162	0.141

Count data are expressed as counts and percentages; continuous data are expressed as mean ± standard deviation or median (interquartile range). Odds ratios are unadjusted. *P* < 0.05 indicates statistical significance.

### Antibiotic use in mothers and infants and the incidence of chorioamnionitis

A comparison was made between maternal and fetal antibiotic use and the incidence of chorioamnionitis. The results showed that the preoperative antibiotic use duration in the L-form positive group was 9.00 (7.00, 10.00) days, significantly longer than the 5.00 (4.00, 6.00) days in the L-form negative group, with a statistically significant difference (*P* < 0.001). This difference in antibiotic exposure itself represents a potential confounder, as differential treatment may have influenced both culture results and clinical outcomes. Regarding the incidence of chorioamnionitis, the overall incidence rate in the L-form positive group was 35.00% (28/80), higher than the 10.00% (4/40) in the L-form negative group (unadjusted OR = 4.85, 95% CI: 1.57–14.95, *P* = 0.004). Among these, the incidence of moderate-to-severe chorioamnionitis in the L-form positive group was 16.25% (13/80), while no severe cases were observed in the L-form negative group. Regarding antibiotic use in offspring, 32 newborns (40.0%) in the L-form positive group received antibiotics for pneumonia or other infection-related indications, including 18 cases treated with broad-spectrum antibiotics (e.g., cephalosporins) and 14 cases treated with targeted antibiotics (selected based on antibiotic susceptibility testing, such as polymyxin, rifampin, or cefazolin). In the L-form negative group, 5 newborns (12.5%) received antibiotics for infections, all of which were broad-spectrum antibiotics. The differences in antibiotic usage rates between the two groups were statistically significant (unadjusted OR = 4.67, 95% CI: 1.67–13.04, χ^2^ = 9.456, *P* = 0.002) (Table 3).

**Table 3:** Antibiotic use in mothers and infants and incidence of chorioamnionitis.

Indicator	L-form positive group (*n* = 80)	L-form negative group (*n* = 40)	Unadjusted OR (95% CI)	Z/t/χ^2^	*P*
Duration of preoperative antibiotic use (days)	9.00 (7.00, 10.00)	5.00 (4.00, 6.00)	–	−6.944	<0.001
Chorioamnionitis (*n*, %)	28 (35.00%)	4 (10.00%)	4.85 (1.57–14.95)	8.523	0.004
Moderate to severe	13 (16.25%)	0 (0.00)	–	–	–
Mild	15 (18.75%)	4 (10.00%)	–	–	–
Antibiotic use rate in newborns (*n*, %)	32 (40.00%)	5 (12.50%)	4.67 (1.67–13.04)	9.456	0.002

Count data are expressed as counts and percentages; continuous data are expressed as median (interquartile range). Odds ratios are unadjusted. *P* < 0.05 indicates statistical significance.

### Exploratory analysis of the microbiological characteristics of bacterial L-forms

The results of exploratory antibiotic susceptibility testing on 80 bacterial L-form positive samples from the L-form positive group showed that bacterial L-forms exhibited high resistance to antibiotics that target cell wall biosynthesis (such as vancomycin, penicillin, and sulfonamides), with sensitivity rates ranging from 7.50% to 10.00%. In contrast, they exhibited apparent sensitivity to antibiotics targeting the cell membrane (e.g., polymyxin, with a sensitivity rate of 82.50%), antibiotics targeting nucleic acids (e.g., rifampin, with a sensitivity rate of 77.50%), and antibiotics inhibiting protein synthesis (e.g., chloramphenicol at 75.00%, lincomycin at 73.75%, erythromycin at 71.25%, tetracycline at 72.50%, aminoglycosides at 70.00%). Additionally, cephalosporin antibiotics such as cefazolin (sensitivity rate 83.75%) and ceftazidime (sensitivity rate 86.25%) exhibited apparent inhibitory effects on bacterial L-forms (Table 4). The observed sensitivity of L-forms to cephalosporins, despite their cell wall-deficient state, has been reported in previous studies (14, 15). However, it must be strongly emphasized that these susceptibility data are exploratory only. No validated susceptibility testing standards or clinical breakpoints exist for L-form bacteria, the detected organisms were not identified to genus or species level, and the clinical relevance of these *in vitro* findings remains entirely uncertain. These results should not be used to guide antibiotic selection in clinical practice without further validation using standardized methods.

**Table 4:** Exploratory analysis of microbiological characteristics of bacterial L-forms.

Types of antibiotics	Mechanism	Sensitivity (%)	Drug resistance rate (%)
Polymyxin	Cell membrane	82.50	17.50
Rifampin	Nucleic acid synthesis	77.50	22.50
Chloramphenicol	Protein synthesis	75.00	25.00
Lincomycin	Protein synthesis	73.75	26.25
Erythromycin	Protein synthesis	71.25	28.75
Tetracycline	Protein synthesis	72.50	27.50
Aminoglycosides	Protein synthesis	70.00	30.00
Vancomycin	Cell wall synthesis	7.50	92.50
Penicillin	Cell wall synthesis	8.75	91.25
Sulfonamide	Folate synthesis	10.00	90.00
Cefazolin	Cell wall synthesis	83.75	16.25
Ceftazidime	Cell wall synthesis	86.25	13.75

### Umbilical cord blood and neonatal peripheral blood L-form bacterial detection

To investigate whether L-form bacteria could be detected in neonatal samples, the presence of L-form bacteria in umbilical cord blood and neonatal peripheral blood was tested. In the L-form positive group, 14 cases (17.5%) of umbilical cord blood L-form bacterial culture were positive, and 12 cases (15.0%) of neonatal peripheral blood were positive. In the L-form negative group, both umbilical cord blood and neonatal peripheral blood L-form bacterial cultures were negative. The detection rates of L-form bacteria in umbilical cord blood and neonatal peripheral blood were higher in the L-form positive group than in the L-form negative group (both *P* < 0.05) (Table 5). While this finding is consistent with the possibility of vertical transmission, several alternative explanations must be considered, including contamination during the sampling process (particularly during cesarean section, when the operative field is exposed), cross-contamination during laboratory processing, or colonization acquired during delivery rather than *in utero*. Molecular confirmation through genotypic matching of isolates between maternal and neonatal samples was not performed in this study, and therefore vertical transmission cannot be confirmed.

**Table 5:** L-form bacterial detection in maternal umbilical cord blood and neonatal peripheral blood.

Sample type	L-form positive group (*n* = 80)	L-form negative group (*n* = 40)	χ^2^	*P*
Umbilical cord blood (*n*, %)	14 (17.50%)	0 (0.00)	7.925	0.005
Peripheral blood of newborns (*n*, %)	12 (15.00%)	0 (0.00)	6.667	0.010

Count data are expressed as numbers and percentages and analyzed using the chi-square test. *P* < 0.05 indicates a statistically significant difference.

### Analysis of the correlation between bacterial L-form detection and intrauterine infection indicators

To further describe the correlation between bacterial L-form detection and intrauterine infection indicators, we used Spearman's correlation coefficient to analyze the relationship between bacterial L-form detection (umbilical cord blood) and intrauterine infection indicators (mean gestational age, mean birth weight, pneumonia infection rate, neonatal mortality rate, and severity of chorioamnionitis) in patients. The results showed that the detection of bacterial L-form was negatively correlated with fetal gestational age (r = −0.334, *P* = 0.003) and birth weight (r = −0.321, *P* = 0.004). Additionally, the detection of bacterial L-form was positively correlated with pneumonia infection rate (r = 0.383, *P* < 0.001), neonatal mortality rate (r = 0.285, *P* = 0.010), and severity of chorioamnionitis (r = 0.319, *P* = 0.004) (Table 6). These correlations are unadjusted and should be interpreted as descriptive associations only; they do not establish causal relationships between L-form detection and adverse outcomes.

**Table 6:** Correlation analysis between bacterial L-form detection and intrauterine infection indicators.

Related indicators	Correlation coefficient (r)	*P*
Gestational age of offspring	−0.334	0.003
Weight of offspring	−0.321	0.004
Pneumonia infection rate	0.383	<0.001
Newborn mortality rate	0.285	0.010
Severity of chorioamnionitis	0.319	0.004

## Discussion

Patients with placenta previa and bleeding experience recurrent hemorrhage due to abnormal placental attachment, which not only weakens the mother's immune defense function but also significantly increases the risk of intrauterine infection. The inflammatory response associated with infection further exacerbates bleeding and pregnancy risks, creating a clinical challenge that is difficult to manage (4, 16, 17). In this process, the phenomenon of negative conventional bacterial cultures despite severe intrauterine infection remains a challenge in obstetric diagnosis and treatment, and the identification of potential pathogens is crucial for improving pregnancy outcomes. Bacterial L-forms, which are cell wall-deficient variants formed by bacteria under adverse conditions, possess characteristics such as latent infection and difficulty in detection by conventional culture (11, 18). However, their role in intrauterine infection in pregnant women with placenta previa and bleeding has not been systematically studied. This exploratory, hypothesis-generating study compared the clinical characteristics, pregnancy outcomes, and microbiological data of patients with and without L-form bacterial detection, and found several descriptive associations that may warrant further investigation. It is essential to note upfront that this study has important limitations—including unresolved confounding, absence of molecular species identification, and reliance on non-validated susceptibility testing—that preclude any conclusions regarding causality, independent pathogenic significance, or therapeutic implications.

The proportions of multiple miscarriages, uterine cavity procedures, and cesarean sections were significantly higher in the L-form positive group than in the L-form negative group, with unadjusted odds ratios ranging from 2.39 to 2.68. While this observation is consistent with the hypothesis that a history of uterine cavity trauma might predispose to bacterial L-form colonization by disrupting the physiological barrier of the endometrium (19, 20), these baseline differences represent critical confounders. Prior miscarriages, uterine procedures, and cesarean sections are each independently associated with intrauterine infection and adverse pregnancy outcomes. Because no multivariable adjustment was performed, the observed associations between L-form positivity and adverse outcomes cannot be separated from the confounding effects of these baseline risk factors. It remains entirely possible that the poorer outcomes observed in the L-form positive group are attributable to their higher burden of uterine trauma history rather than to L-form infection *per se*.

The average gestational age and birth weight of offspring from the L-form positive group were lower, and the pneumonia infection rate was higher (32.5% vs. 7.5%, unadjusted OR = 5.93). Additionally, the incidence of chorioamnionitis (35.00%) was higher in the L-form positive group compared to the L-form negative group (10.00%, unadjusted OR = 4.85). While these findings are consistent with the hypothesis that L-form bacterial detection may be associated with adverse pregnancy outcomes, the lack of multivariable adjustment means that the independent contribution of L-form detection to these outcomes, beyond the confounding effect of uterine trauma history and other baseline differences, cannot be determined from this study. While the neonatal mortality rate in the L-form positive group (10.00%) was numerically higher than that in the L-form negative group (2.50%), the difference did not reach statistical significance (unadjusted OR = 4.33, 95% CI: 0.52–36.00, *P* = 0.141), likely due to insufficient statistical power for this rare outcome.

L-form bacteria were detected in umbilical cord blood (17.5%) and neonatal peripheral blood (15.0%) in the L-form positive group, while both were negative in the L-form negative group. While these findings raise the possibility of maternal-fetal transfer or shared exposure, contamination during sampling and processing cannot be fully excluded despite the use of duplicate inoculation, matched negative controls, and repeat reading. Therefore, the neonatal detection results should be regarded as exploratory evidence rather than definitive proof of vertical transmission.

Exploratory susceptibility testing showed that bacterial L-forms exhibited low sensitivity to cell wall-active antibiotics (7.50%−10.00%) but apparent sensitivity to polymyxin (82.50%), rifampin (77.50%), protein synthesis inhibitors (70.00%−75.00%), and cephalosporins (83.75%−86.25%). These findings must be interpreted with extreme caution and should not be used to guide clinical antibiotic selection for several important reasons. First, no validated susceptibility testing standards or clinical breakpoints exist for L-form bacteria; our modified CLSI disk diffusion methodology was adapted from protocols designed for walled bacteria and may not accurately reflect the *in vivo* susceptibility of L-forms. Second, without genus- or species-level identification, the susceptibility profiles cannot be contextualized within established resistance patterns for any particular organism. Third, the apparent sensitivity to cephalosporins—seemingly paradoxical for cell wall-deficient bacteria—has been reported in previous studies (14, 15) but may reflect the presence of unstable L-forms or spheroplasts with residual cell wall material that partially revert during susceptibility testing (21, 22), the heterogeneous nature of clinical L-form populations, or artifacts of the testing methodology itself. Fourth, the possibility that some detected L-forms represent culture-induced variants rather than true *in vivo* cell wall-deficient bacteria cannot be excluded, which would further limit the relevance of susceptibility data. These susceptibility results are presented purely as descriptive, exploratory observations and serve only to generate hypotheses for future investigation using validated methods.

The finding that the L-form positive group required longer preoperative antibiotic use (median 9 days vs. 5 days) is notable but introduces additional interpretive complexity. Differential antibiotic exposure before sampling may have influenced culture positivity rates (by selecting for L-form conversion) and may have independently affected clinical outcomes, representing a further confounder in this observational study.

## Limitations

This study has several important limitations that substantially constrain the interpretation of its findings. First, this was a single-center retrospective observational study, which introduces selection bias and limits generalizability. Second, and most critically, the L-form positive cohort had significantly higher rates of prior uterine trauma (miscarriages, uterine procedures, and cesarean sections), which are well-established independent risk factors for intrauterine infection and adverse pregnancy outcomes. No multivariable regression analysis was performed to adjust for these confounders, and therefore the observed associations between L-form detection and adverse outcomes cannot be attributed to L-form infection independently of these baseline differences. The unadjusted odds ratios presented throughout this study should be interpreted as descriptive measures only. Third, bacterial L-forms were identified primarily based on culture morphology and growth characteristics without molecular confirmation using 16S rRNA sequencing or other validated molecular methods. Consequently, the detected L-forms were not characterized to genus, species, or even reliable Gram classification, which severely limits interpretation of their clinical significance and the meaning of susceptibility data. Fourth, the possibility of stress- or antibiotic-induced L-form conversion during culture cannot be excluded; some or all of the detected L-forms may represent *in vitro* artifacts rather than organisms that existed *in vivo* in the L-form state. Fifth, the susceptibility testing was performed using modified CLSI disk diffusion methodology, but no validated interpretive criteria or breakpoints exist for L-form bacteria, rendering these results exploratory and unsuitable for clinical decision-making. Sixth, the detection of L-forms in neonatal samples may reflect contamination or peripartum acquisition rather than true vertical transmission, and molecular matching of maternal and neonatal isolates was not performed. Seventh, differential antibiotic exposure between groups before sampling may have affected both culture positivity rates and outcome comparisons. Eighth, we did not measure maternal cytokine levels, which would have helped characterize the inflammatory milieu. Ninth, the study was underpowered for the neonatal mortality outcome. Finally, Trial Group 1 and Trial Group 2 were analyzed as a combined L-form positive group, and separate analyses or sensitivity analyses stratifying by timing of detection were not performed.

## Future directions

Future studies should employ prospective multi-center designs with larger sample sizes and, crucially, should utilize molecular methods (16S rRNA sequencing or metagenomic analysis) for definitive species identification and confirmation of vertical transmission through genotypic matching of maternal and neonatal isolates. Validated MIC-based susceptibility testing methods for L-forms should be established before any therapeutic implications are drawn. Multivariable analyses adjusting for baseline differences including uterine trauma history would be essential to determine whether L-form detection contributes independently to adverse outcomes. Separate analyses of patients with early vs. late L-form detection (corresponding to Trial Groups 1 and 2) would help clarify the temporal relationship between detection and outcomes. Additionally, quality control measures including QC strains for L-form susceptibility testing and negative controls to assess contamination rates should be incorporated into future study designs.

In summary, this hypothesis-generating study found descriptive associations between bacterial L-form detection and markers of intrauterine infection in pregnant women with placenta previa and bleeding. L-form positivity was observed more frequently in patients with a history of uterine cavity trauma and was associated with higher unadjusted rates of chorioamnionitis, preterm birth, and neonatal pneumonia. However, due to significant baseline confounding, absence of molecular species identification, reliance on non-validated susceptibility testing, and the inability to confirm the *in vivo* biological relevance of detected L-forms, no conclusions regarding causality, independent pathogenic significance, or therapeutic implications can be drawn from this study. These findings serve only to generate the hypothesis that bacterial L-forms may play a role in occult intrauterine infections in this high-risk population, a hypothesis that requires rigorous testing in future prospective studies with appropriate microbiological and statistical methodology.

## Data Availability

The original contributions presented in the study are included in the article/supplementary material, further inquiries can be directed to the corresponding author.
